# Epstein-Barr Virus BBRF2 Is Required for Maximum Infectivity

**DOI:** 10.3390/microorganisms7120705

**Published:** 2019-12-16

**Authors:** H. M. Abdullah Al Masud, Yusuke Yanagi, Takahiro Watanabe, Yoshitaka Sato, Hiroshi Kimura, Takayuki Murata

**Affiliations:** 1 Department of Virology, Nagoya University Graduate School of Medicine, Nagoya 466-8550, Japan; 2 Department of Microbiology, Faculty of Biological Sciences, University of Chittagong, Chattogram 4331, Bangladesh; 3 Department of Virology and Parasitology, Fujita Health University School of Medicine, Toyoakev 470-1192, Japan

**Keywords:** EBV, BAC, CRISPR/Cas9, BBRF2, lytic cycle

## Abstract

Epstein-Barr virus (EBV) is a member of the gammaherpesvirinae, which causes infectious mononucleosis and several types of cancer. BBRF2 is an uncharacterized gene of EBV and is expressed during the lytic phase. To evaluate its function, BBRF2-knockout EBV was prepared using bacterial artificial chromosome (BAC) technology and the CRISPR/Cas9 system. Although viral gene expression, DNA synthesis, and progeny secretion were not affected, the infectivity of progeny viruses was significantly reduced by the disruption of BBRF2. When expressed alone, BBRF2 protein localized to the nucleus and cytoplasm, while the coexpression of an interacting partner, BSRF1, resulted in its relocalization to the cytoplasm. Interestingly, the coexpression of BBRF2 protected BSRF1 from proteasome/ubiquitin-dependent degradation. Therefore, BBRF2, together with BSRF1, augments viral infectivity.

## 1. Introduction

Among the Herpesviridae family, Epstein-Barr virus (EBV) is a ubiquitous human virus that can cause infectious mononucleosis and cancers, such as Burkitt lymphoma, Hodgkin lymphoma, T/NK cell lymphoma, and nasopharyngeal carcinoma [1,2]. Being a member of the gammaherpesvirinae, it has a large (~175 kb), double-stranded DNA genome that encodes more than 80 genes.

Like other herpesviruses, the lifecycle of EBV comprises latent and lytic phases. In the latent phase, EBV genomes are maintained as episomes in the nucleus; during this stage, a few latent genes, such as EBV nuclear antigen 1 (*EBNA1*) and latent membrane protein 1 (*LMP1*) [3], are expressed. The reactivation of the virus results in the expression of all lytic genes in a cascade manner [4]. First, a subset of viral genes, *BZLF1* (also known as *Zta*, *ZEBRA*, or *EB1*) and *BRLF1* (*Rta*), is expressed. These are classified as immediate–early genes, because of the rapidity of their expression. These transcriptional activators induce the expression of early viral genes, including factors required for viral DNA genome synthesis, such as viral DNA polymerase (*BALF5*), accessory factor of the polymerase (*BMRF1*), and single-stranded DNA-binding protein (*BALF2*). After viral DNA replication, the transcription of viral late genes takes place. The late genes encode structural proteins of the progeny virus, such as capsid proteins, tegument proteins, and glycoproteins. It is assumed that viral nucleocapsids are formed in the nucleus and then bud into the nuclear membrane (known as initial envelopment) to leave the nucleus. Next, the nucleocapsids gain tegument proteins and enter a membranous organelle in the cytoplasm, likely the *trans*-Golgi network or endosomes; this process is termed “secondary envelopment”. Finally, the mature progeny virions are released from the host cell.

Several EBV lytic genes have yet to be studied [5], including *BBRF2*, which is conserved among all herpesvirus families and encodes a protein of 278 amino acids. The *BBRF2* gene is a homolog of herpes simplex virus 1 (HSV-1) *UL7,* cytomegalovirus (CMV) *UL103*, and Kaposi sarcoma-associated herpesvirus (KSHV) *ORF42* [5]. Because its homologs in other herpesviruses are tegument proteins, EBV BBRF2 is also thought to be a tegument component, although the protein has not been detected in virus particles, possibly due to its low abundance [6].

It has been reported that the UL7 gene product of alphaherpesviruses, including HSV-1, is a tegument component that directly interacts with the UL51 protein [7,8]. The UL7 homologs are dispensable for viral replication, but are assumed to play important roles in progeny virion formation, egress, cell-to-cell spread, and cell morphology [7,8,9,10,11]. A deletion mutant in the human CMV *UL103* gene exhibited reduced maturation of virus particles, possibly due to the inhibition of viral egress from infected cells [12]. Screening using small interfering RNAs (siRNAs) identified CMV *UL103* as a crucial gene for cytoplasmic virion assembly [13]. Also, the KSHV *ORF42* gene is reportedly required for efficient virion production, as well as viral gene expression [14]. Mutagenesis of murine gamma-herpesvirus-68 (MHV-68) revealed that its *ORF42* gene was essential for virus replication [15].

The BBRF2 gene product of EBV interacts with BSRF1 protein, the homolog of HSV UL51 [16]. Because alphaherpesvirus UL51 is a palmitoylated protein that associates with membranes and accumulates in the Golgi apparatus, UL51 (and possibly also its homologs, including EBV BSRF1) is presumed to function in secondary envelopment in the cytoplasm [17,18,19].

We prepared *BBRF2*-knockout EBV by means of bacterial artificial chromosome (BAC) technology and the CRISPR/Cas9 system. The infectivity of the mutant virus was significantly lower than that of wild-type and revertant viruses. Also, the intracellular localization of BBRF2 protein changed to a Golgi-like pattern upon coexpression of BSRF1, providing solid evidence for a BBRF2-BSRF1 interaction. Furthermore, expression of the BBRF2 gene product protected BSRF1 protein from proteasome-dependent degradation. Overall, we demonstrated that BBRF2 of EBV plays a similar role to its counterparts in other herpesviruses.

## 2. Materials and Methods

### 2.1. Cell Culture and Reagents

HeLa, HEK293, HEK293T, and HEK293 EBV-BAC cells were cultured in Dulbecco’s modified Eagle’s medium (Sigma-Aldrich, St. Louis, MO, USA) supplemented with 10% fetal bovine serum (FBS). Akata(−) cells and recombinant Akata virus-containing AGS/EGFP-EBV cells harboring EGFP and G418 resistance genes [20] were cultured in Roswell Park Memorial Institute 1640 medium (Sigma-Aldrich) containing 10% FBS.

The antibodies against BRLF1, BMRF1, BALF2, BALF5, BGLF4, gB (BALF4), BKRF4, BOLF1, and BSRF1 have been described previously [16,21,22,23,24,25]. Mouse anti-FLAG, mouse anti-BMRF1, rabbit anti-α/β-tubulin, and rat/rabbit anti-HA antibodies were purchased from Sigma-Aldrich, Novocastra (Weltzlar Germany), Cell Signaling Technology (Danvers, MA, USA), and Roche (Basel Switzerland), respectively. Horseradish peroxidase-conjugated secondary mouse, rabbit, and rat IgGs were purchased from Amersham Biosciences (Little Chalfont UK). The Alexa 488- and Alexa 546-conjugated goat anti-rabbit and anti-mouse secondary IgGs were obtained from Molecular Probes (Eugene, OR USA). MG132 and Bortezomib were purchased from Sigma-Aldrich.

### 2.2. Expression Plasmids

The BZLF1, FLAG-tagged BKRF4, FLAG/HA-tagged BSRF1, and HA-tagged BBRF2 expression plasmids have been described previously [16,24,25,26]. A stop codon was inserted into the *BBRF2* sequence and mutated by inverse PCR using the primers forward 5′-ATGCGGCTACGTCCTCGTGAG-3′ and reverse 5′-TACTGGGACGAGATCATCCGG-3′. The HA-tagged BBRF2 vector was used as the template.

The *BBRF2* expression vector (pcDNABBRF2) was constructed by inserting the BBRF2 ORF into pcDNA3 (Invitrogen) using the primers forward 5′-TAGAGAATTCATGGCATCCGGCAAGCAC-3′ and reverse 5′-CTATCTCGAGCTAGGGAATTATTTTTGAGAC-3′. The N-terminal FLAG-tagged BBRF2 expression vector (pcDNAFLAGBBRF2) was constructed by inserting the BBRF2 ORF into pcDNAFLAG.

To prepare CRISPR/Cas9-mediated BBRF2-knockout, we constructed pX459-BBRF2, in which two oligonucleotide sequences (forward 5′-CACCGCACTCCAAGTGCAACAATC-3′ and reverse 5′-AAACGATTGTTGCACTTGGAGTGC-3′) were annealed and inserted into the *Bbs*I site of pX459 (Addgene, USA), a vector for CRISPR/Cas9. Similarly, we prepared a second pX459-BBRF2 using the primers forward 5′-CACCGACCCAACAATGTGAACCTTA-3′ and reverse 5′-AAACTAAGGTTCACATTGTTGGGTC-3′. Finally, the mutation was assessed using the primers forward 5′-TTAACCGCCATAACGCCATC-3′ and reverse 5′-CCTTGTTTAGATCCGCGCAGT-3′.

### 2.3. BBRF2 Knockout by Genetic Manipulation of the EBV-BAC Genome and Establishment of HEK293-Cell Clones

The EBV-BAC DNA (B95-8 strain) was provided by W. Hammerschmidt [27]. To construct the *BBRF2* knock-out EBV-BAC genome, homologous recombination was carried out in *Escherichia coli*, as described elsewhere [25]. The Neo/St cassette, which contains neomycin-resistance and streptomycin-sensitivity genes, was used as a selection marker. The recombination vectors were constructed by PCR using the rpsL-neo vector (Gene Bridges, Heidelberg, Germany) as the template and the following primers: Neo/St forward 5′-GGGACCTTAACATCCGAGACCTCCGGGCCCACGTCAAGGCCCGGATGATCTCGTCCCAGTGGCCTGGTGATGATGGCGGGATC-3′ and Neo/St reverse 5′-TATGTTGAGGTGGTCGACCTGGTCCTCGGAGTCCAGCAGACTCACGAGGACGTAGCCGCATCAGAAGAACTCGTCAAGAAGG-3′. After recombination, the insertion mutant (intermediate #1) was screened by antibiotic selection and confirmed by colony PCR using the primers forward 5′-AAAAAGTAAGCCTGCGCGTG-3′ and reverse 5′-GAGGGGCTCTTGGCATTCTC-3′. The stop codon-containing mutant (dBBRF2-stop) was constructed by replacing the Neo/St cassette with the mutated EBV sequence, followed by streptomycin selection. To prepare the revertant virus (dBBRF2-stop/R), the Neo/St cassette was inserted into the dBBRF2-stop strain (intermediate #2), and the cassette was replaced with the wild-type EBV sequence. Electroporation was conducted using a Gene Pulser III (Bio-Rad, Hercules, USA), and the EBV-BAC DNAs were purified using NucleoBond Bac100 (Macherey–Nagel, Düren, Germany). After construction of recombinant EBV-BAC strains, the DNAs were digested with BamHI or EcoRI and resolved by agarose gel electrophoresis.

HEK293 cells were transfected with EBV-BAC DNA using Lipofectamine 2000 reagent (Invitrogen, USA) followed by hygromycin selection, and green fluorescent protein (GFP)-positive cell colonies were selected for preparation of cell clones.

### 2.4. Preparation of BBRF2-Knockout Akata Virus Using the CRISPR/Cas9 System

Akata *BBRF2* knockout (BBRF2-KO) virus was prepared as described previously [24]. In brief, pX459-BBRF2 was transfected into AGS/EGFP–EBV (containing recombinant Akata virus) using Lipofectamine 2000 reagent. Puromycin-resistant cells were selected and transfected with the BZLF1 expression vector to induce progeny production. The virus-containing cell-free supernatants were collected and used to infect Akata(−) cells. GFP-positive, geneticin G418 (750 µg/mL)-resistant cell clones were prepared by limited dilution.

### 2.5. Lytic Induction, Immunoblotting, ebv dna Quantification, and Viral Titer Determination

HEK293 and Akata cells harboring the latent EBV genome were lytically induced by transfection of the BZLF1 expression vector by electroporation (Neon Transfection System; Thermo Fisher Scientific, Waltham, MA, USA), and by adding an anti-IgG antibody, respectively.

Immunoblotting was performed as described previously [25]. At two days posttransfection, cells were washed with phosphate-buffered saline (PBS), harvested, and solubilized in sample buffer by sonication and heat treatment. The samples were subjected to sodium dodecyl sulfate-polyacrylamide gel electrophoresis, followed by immunoblotting.

The EBV DNA level was quantified using the Fast Start Universal Probe Master (Roche Applied Science, Penzberg, Germany), as reported previously [28]. Briefly, cells were washed with PBS, lysed by sonication in lysis buffer, treated with proteinase K and, after heat-inactivation of proteinase K, subjected to qPCR analysis. The standard curve, primers, and probe used for quantifying EBV DNA are described elsewhere [28].

To assay the cell-free virus level, the culture supernatants were treated with Turbo DNase I (Thermo Fisher Scientific) to eliminate naked EBV DNA and subjected to DNA extraction using a DNeasy Blood and Tissue Kit (Qiagen, Hilden, Germany). Extracellular EBV DNA was quantified by qPCR, as described previously [24].

For infectivity assays, culture supernatants of HEK293 cells were collected, centrifuged, and filtered after three days of BZLF1 transfection. For Akata cells, supernatants were collected at two days after addition of an anti-IgG antibody, followed by centrifugation and filtration. Next, the supernatants containing virus particles were cocultured with Akata(−) cells with rotation at room temperature for 3 h, and centrifuged at low speed; the pellets were resuspended in fresh medium and cultured for two days. The cells were fixed in 1% formaldehyde and washed in PBS, and the percentage of GFP-positive cells was determined using the FACSCalibur G5 system (Becton Dickinson, Franklin Lakes, NJ, USA).

### 2.6. Immunoprecipitation Analysis

Immunoprecipitation was carried out as described elsewhere [23]. Briefly, HEK293T cells were transfected using Lipofectamine 2000 reagent. At 24 h posttransfection with the indicated expression vectors, the cells were suspended in lysis buffer, followed by sonication and high-speed centrifugation. Mouse anti-FLAG/anti-HA antibodies and G-Sepharose 4 Fast Flow (GE Healthcare, Chicago, IL, USA) were added to the supernatants and the mixtures were incubated with rotation at 4 °C. Next, the immunocomplexes were washed, mixed with sample buffer, and subjected to immunoblotting with the indicated antibodies. The protocol for the ubiquitination assay was described previously [29].

### 2.7. Immunofluorescence Analysis

Immunofluorescence analysis was conducted as described previously [24]. HeLa cells were transfected with the expression plasmids (pcDNA, pcDNA+FLAG-BBRF2, pcDNA+BSRF1-HA, and FLAG-BBRF2+BSRF1-HA). At one day posttransfection, cells were processed and treated with the primary antibodies, as described previously. Next, the cells were treated with the secondary antibodies followed by washing in PBS. Samples were mounted in ProLong Gold antifade reagent with DAPI (Molecular Probes) and examined using an LSM880 confocal microscope (Zeiss, Oberkochen, Germany).

## 3. Results

### 3.1. Knockout of the BBRF2 Gene in EBV-BAC

**Figure 1:**
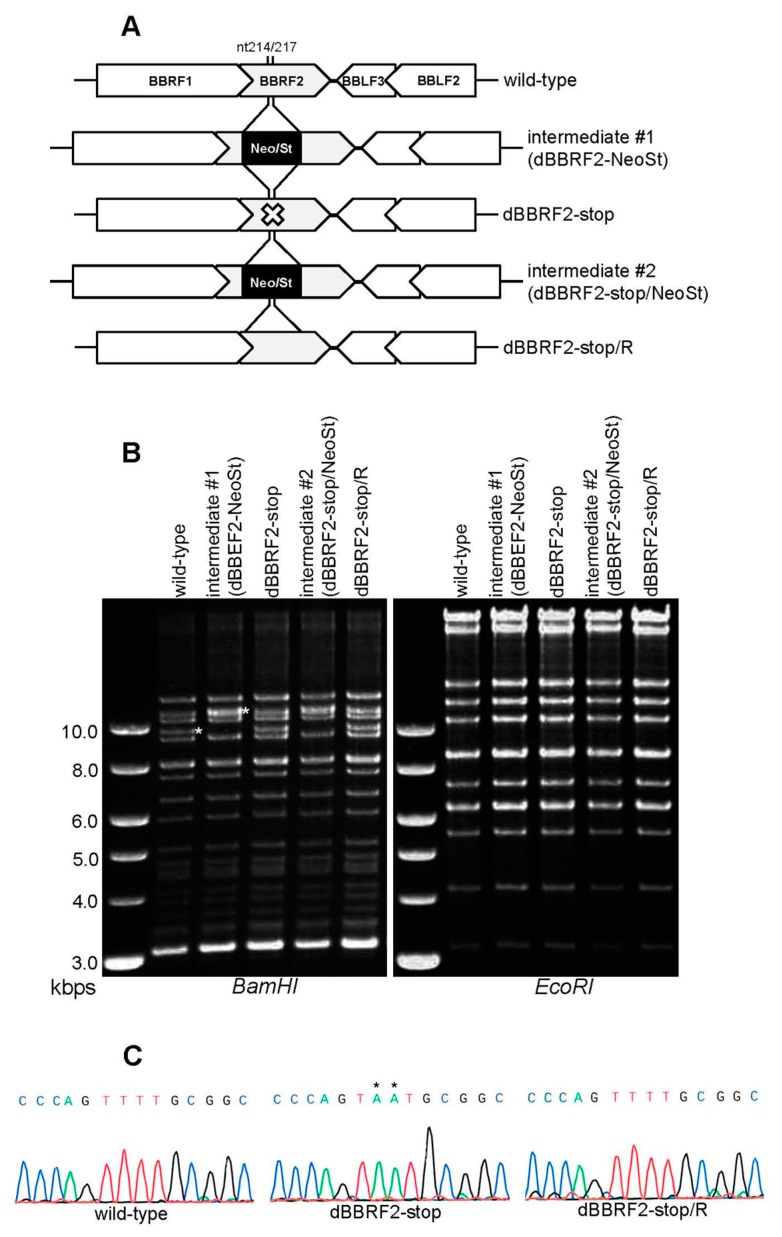
Construction of Epstein-Barr virus (EBV) *BBRF2* mutant and revertant viruses using the EBV-bacterial artificial chromosome (BAC) system. (**A**) Diagram of BBRF2-deficient virus construction by EBV-BAC recombination in *E. coli*. The Neo/St cassette, containing neomycin-resistance and streptomycin sensitivity genes (black box), was inserted between nucleotides 214 and 217 of the *BBRF2* gene to prepare an intermediate (#1), and was replaced with the *BBRF2* sequence containing a stop codon (TTT to TAA) to construct the *BBRF2*-deficient mutant (dBBRF2-stop). To produce revertant virus (dBBRF2-stop/R) from its corresponding mutant, the cassette was again inserted and replaced with the wild-type BBRF2 sequence. (**B**) Recombinant EBV-BAC DNAs were digested with *Bam*HI or *Eco*RI and subjected to agarose gel electrophoresis. The white asterisk in the *Bam*HI-digested wild-type genome indicates the *Bam*HI-B fragment, which contains the *BBRF2* gene; the asterisk in the intermediate #1 sample indicates the size of the *Bam*HI-B fragment with the Neo/St cassette. (**C**) Sequence data of the recombinant viruses.

Analyzing the phenotype of a knockout virus enables functional analysis of genes, but no *BBRF2*-knockout EBV strain has been reported. Therefore, we prepared *BBRF2*-knockout virus using the EBV-BAC system (Figure 1). By inserting and removing a selection marker cassette (Neo/St) in bacteria, a stop codon was introduced in the N-terminal region (nt 214/217) of the *BBRF2* gene (Figure 1A, dBBRF2-stop). The repaired strain from the stop mutant was also prepared (Figure 1A, dBBRF2-stop/R). Agarose gel electrophoresis was performed after *Bam*HI or *Eco*RI digestion to verify recombination (Figure 1B). A DNA band of about 10 kb, containing the *BBRF2* gene, in the *Bam*HI-digested pattern migrated slowly in the intermediate strain (intermediate #1) due to the cassette (Figure 1B, left panel). The *Eco*RI-digestion pattern was almost identical between the strains (Figure 1B, right panel). The sequences of the recombinant viruses were confirmed (Figure 1C).

After the purification of recombinant BACs, HEK293 cells were transfected with the EBV-BAC DNAs, followed by hygromycin selection. Typical hygromycin-resistant and GFP-positive cell clones were isolated and used in subsequent experiments.

### 3.2. Disruption of BBRF2 Decreased Infectivity

**Figure 2:**
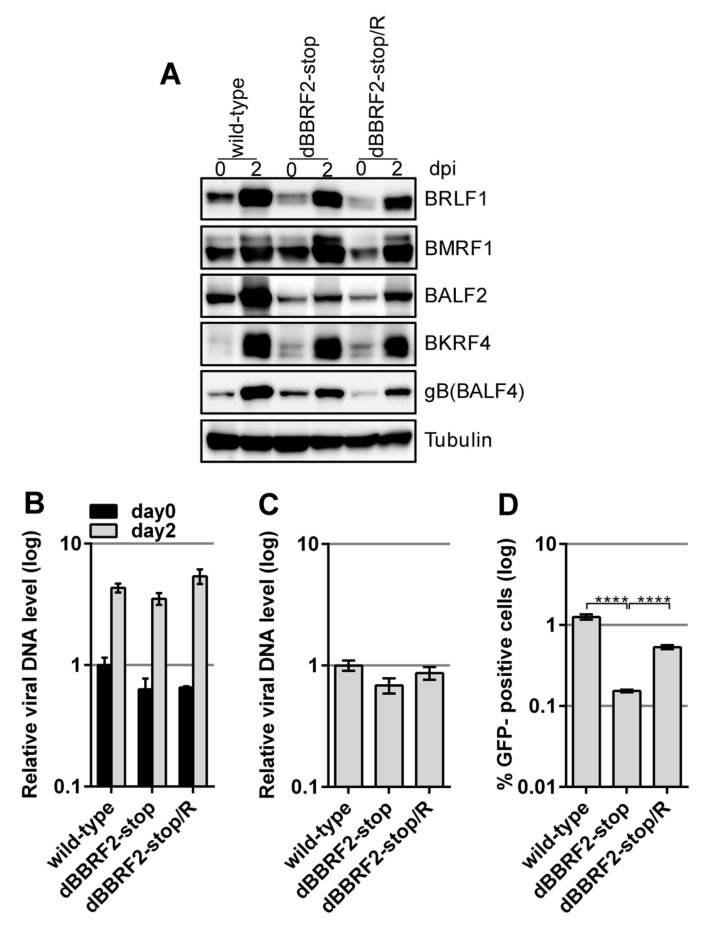
Disruption of the EBV *BBRF2* gene significantly reduced viral infectivity in HEK293 cells. Recombinant EBV-BAC DNAs (Figure 1) were transfected into HEK293 cells and green fluorescent protein (GFP)-positive cell clones were isolated after hygromycin selection. (**A**) EBV protein levels in HEK293 cells. The EBV lytic cycle was induced by electroporation of an expression vector carrying the immediate-early gene, BZLF1. Cells were harvested immediately (day 0) and at two days after transfection (day two), and subjected to immunoblotting with the indicated antibodies. (**B**) Viral DNA synthesis in HEK293 cells. Total DNA was extracted from cells at zero or two days after BZLF1 transfection and subjected to qPCR. Means ± SD of three independent biological replicates are shown after normalization to the host genomic DNA level. (**C**) Extracellular progeny production from HEK293 cells. Cell-free medium was collected three days after BZLF1 transfection, followed by DNaseI treatment to eliminate naked viral DNA. DNA was then extracted and subjected to qPCR. Each bar represents the mean ± SD DNA level of three independent biological replicates. (**D**) Infectivity of progeny virus. Aliquots of the supernatants used in (C) were infected into Akata(–) cells. Two days after infection, GFP-positive cells were assayed by FACS. Each bar represents the mean ± SD infectivity of three independent biological replicates. **** *p* < 0.0001, Student’s *t*-test.

After preparing the *BBRF2*-knockout virus and establishing HEK293 cells with latent virus, we next examined whether the lytic cycle of EBV was influenced by the mutation. The lytic viral cycle was induced by transfecting the BZLF1-expression vector into HEK293 cells containing latent recombinant EBV (Figure 2). The expression levels of immediate early (BRLF1), early (BMRF1 and BALF2), and late (BKRF4 and gB(BALF4)) viral proteins were similar at two days after BZLF1 transfection (Figure 2A). Although the levels of BALF2 and gB(BALF4) in the knockout strain (dBBRF2-stop) were lower than those in the wild-type, they were also lower in the revertant strain (dBBRF2-stop/R), suggesting that the differences are caused by clonal or technical variations. Therefore, *BBRF2* does not play a role in viral gene expression in HEK293 cells. The introduction of a stop mutation into the *BBRF2* gene resulted in little or no effect on viral DNA synthesis at day two (Figure 2B). The titer of DNase-resistant progeny virus in the medium was monitored by qPCR (Figure 2C). At three days after BZLF1 transfection, the medium was collected and subjected to low-speed centrifugation and filtration. DNase was added to the medium to eliminate naked DNA and inappropriately-packaged viral DNA, and DNA in the medium was extracted and quantified. The secretion of DNase-resistant virus, i.e., properly packaged virus DNA, was not affected by mutation of the *BBRF2* gene (Figure 2C). However, the infectivity of *BBRF2*-knockout virus in the same medium was significantly lower (Figure 2D), indicating that the *BBRF2* gene is required for maximum infectivity.

### 3.3. Knockout of the BBRF2 Gene by CRISPR/Cas9

**Figure 3:**
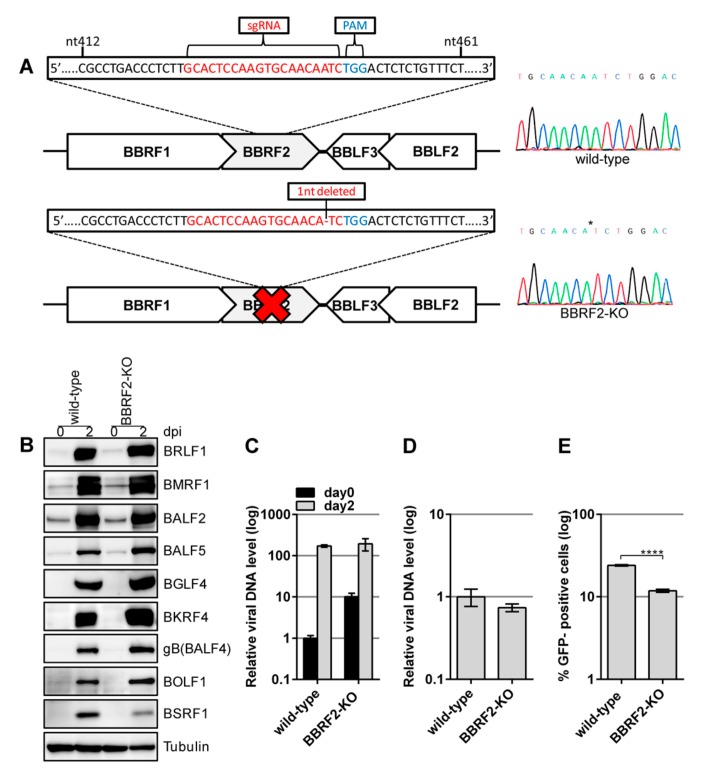
*BBRF2* knockout (BBRF2-KO) in Akata virus using the CRISPR/Cas9 system reduced progeny viral infectivity. (**A**) Schematic of BBRF2-KO in Akata EBV. AGS/EGFP–EBV cells were transfected with the CRISPR/Cas9 vector, pX459-BBRF2. After puromycin selection, progeny virus was infected into Akata(–) cells. The Akata cells latently infected with BBRF2-KO EBV were cloned in the presence of G418, followed by sequencing. Wild-type (upper panel) and edited (lower panel) sequences are shown. (**B**) Viral protein levels in Akata cells. Cells were harvested at day zero and two after anti-IgG treatment and subjected to immunoblotting with the indicated antibodies. (**C**) EBV DNA synthesis in Akata cells. Samples were prepared as in Figure 2B and subjected to qPCR. (**D**) Extracellular progeny. production from Akata cells. Akata cell clones (wild-type and BBRF2-KO) were treated with the anti-IgG antibody for two days, and the culture supernatants were collected and processed as described in Figure 2C. (**E**) Infectivity of progeny virus. A portion of the culture supernatants prepared in (D) was used to infect Akata(–) cells and the frequency of GFP-positivity was determined by FACS. Means ± SD of three independent biological replicates are shown. **** *p* < 0.0001, Student’s *t*-test.

Disruption of the *BBRF2* gene in the B95-8 strain of EBV-BAC indicated that it is involved in progeny virion maturation. A second strain (Akata) of *BBRF2*-knockout EBV was generated using the CRISPR/Cas9 system (Figure 3). An sgRNA was used to target the central part of the *BBRF2* gene (Figure 3A). AGS cells latently infected with recombinant Akata EBV were transfected with the CRISPR vector, and the lytic cycle was induced. The progeny virus (a mixture of edited and nonedited viruses) was infected into Akata(–) cells, followed by limiting dilution and cloning in the presence of G418. A sequencing analysis confirmed that one of the cell clones contained EBV harboring a *BBRF2* gene with a one-nucleotide deletion (Figure 3A, BBRF2-KO).

Reactivation was induced by anti-IgG antibody treatment. The levels of viral proteins were similar among genes, although that of BSRF1 was lower (Figure 3B). Viral DNA synthesis after the induction (Figure 3C) and production of DNase-resistant virions (Figure 3D) were not affected by the *BBRF2* mutation in Akata cells, but the infectivity of the virus was significantly reduced by BBRF2-KO (Figure 3E).

**Figure 4:**
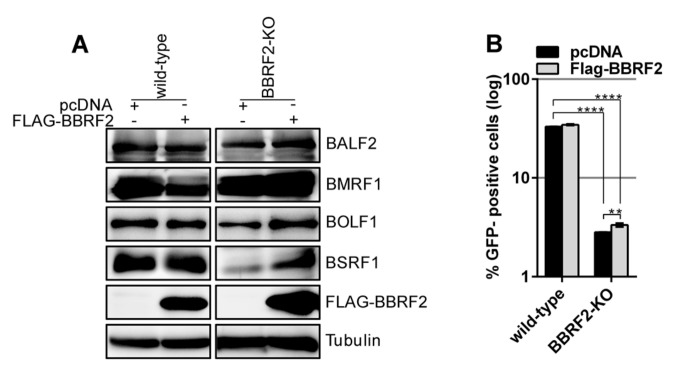
Complementation of reduced progeny infectivity in BBRF2-KO. (**A**) Exogenous supply of BBRF2 in Akata BBRF2-KO cells. The FLAG-BBRF2 expression plasmid or its empty pcDNA vector was electroporated into the Akata wild-type and *BBRF2*-knockout cells prepared in Figure 3. The cells were cultured in the presence of an anti-IgG antibody and harvested after two days for immunoblotting. (**B**) Exogenous expression of BBRF2 only marginally restored the infectious progeny titer. Cell-free culture supernatants were collected from the samples in (A) and infected to Akata(–) cells. Infectious virions were determined by enumerating GFP-positive cells by FACS. Each bar represents the mean ± SD titer of three independent biological replicates. ** *p* < 0.005 and **** *p* < 0.0001, Student’s *t*-test.

We next tested whether the exogenous expression of BBRF2 could restore the infectivity of the BBRF2-KO. Protein levels were determined by western blotting (Figure 4A). The exogenous supply of a BBRF2 expression vector yielded significant, albeit marginal, restoration of infectivity (Figure 4B). The reason why exogenous expression of BBRF2 did not fully complement the titer of BBRF2-KO is unknown.

### 3.4. BBRF2 Was Recruited to the Cytoplasm by BSRF1

**Figure 5:**
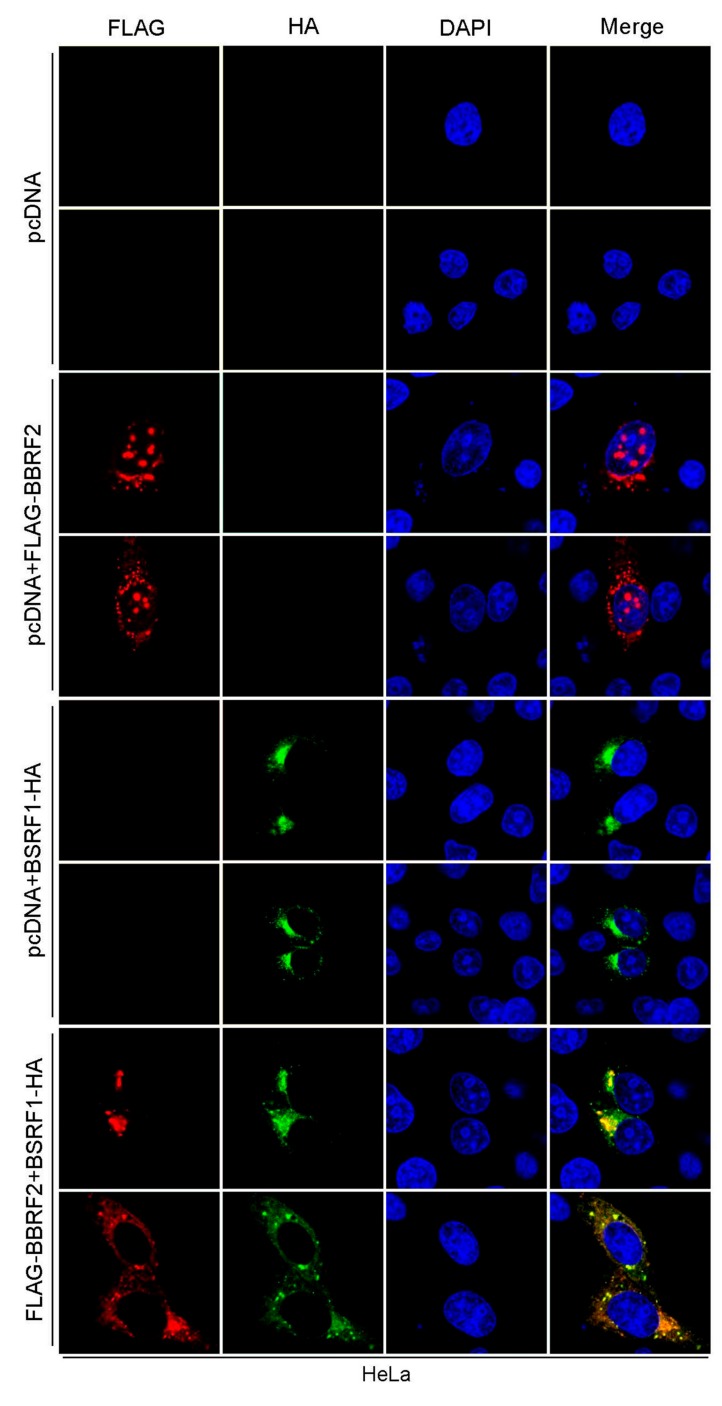
BBRF2 localized to the nucleus and cytoplasm in a punctate pattern, but completely re-localized to the cytoplasm upon cotransfection with its interaction partner BSRF1. HeLa cells were transfected with the indicated expression vectors, fixed at one day after transfection, stained with anti-FLAG (red) and anti-HA (green) antibodies and DAPI (blue), and visualized by confocal laser microscopy at 63 × (2.7 × zoomed).

Next, the intracellular localization of *BBRF2* was examined by immunofluorescence. We first attempted to stain the endogenous *BBRF2* gene product in infected cells but were unsuccessful due to issues with the BBRF2 antiserum. Thus, we analyzed the localization in transfected cells (Figure 5). BBRF2 protein was found in both the nucleus and the cytoplasm in a discrete, dot-like pattern (Figure 5, pcDNA + FLAG-BBRF2). We recently reported that BBRF2 (homolog of HSV UL7) interacts with BSRF1 (that of HSV UL51) [16]; thus, we analyzed the effect of BSRF1 cotransfection on the distribution of BBRF2. BBRF2 protein that is predominantly localized to the cytoplasm with coexpression of BSRF1 (Figure 5, FLAG-BBRF2 + BSRF1-HA). Because BSRF1 localizes to the Golgi apparatus when expressed alone [16] (Figure 5, pcDNA+BSRF1-HA), BBRF2 protein was likely recruited to the Golgi apparatus by BSRF1. This result accords with that for HSV; i.e., HSV UL7 associates with UL51, which is responsible for cytoplasmic recruitment of UL7 [8].

### 3.5. Association of BBRF2 with Another Tegument Component, BGLF2

**Figure 6:**
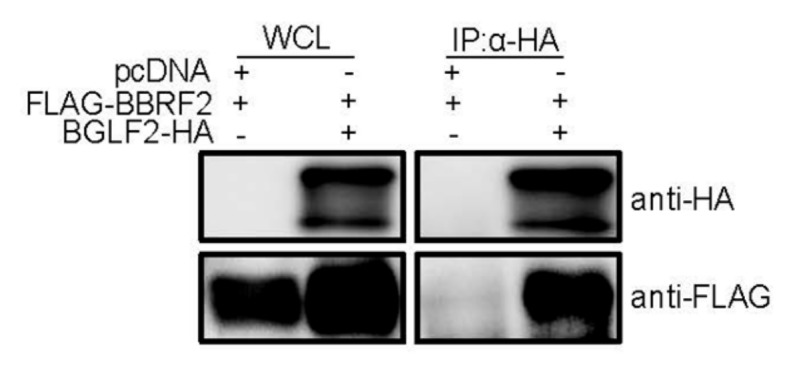
BBRF2 interacted with the EBV tegument protein, BGLF2. HEK293T cells were cotransfected with the indicated expression plasmids. After 24 h, whole-cell lysates (WCLs) were collected and subjected to immunoblotting (left panels). A portion of the cell lysates was subjected to immunoprecipitation using an anti-HA antibody, followed by immunoblotting (right panels).

In addition to BSRF1, a tegument protein, BGLF2, coprecipitated with the BBRF2 gene product (Figure 6). The BBRF2 homolog in HSV, UL7, reportedly interacts with UL14, which, in turn, associates with the BGLF2 homolog, UL16 [30]. 

### 3.6. Stabilization of BSRF1 Protein by BBRF2

**Figure 7:**
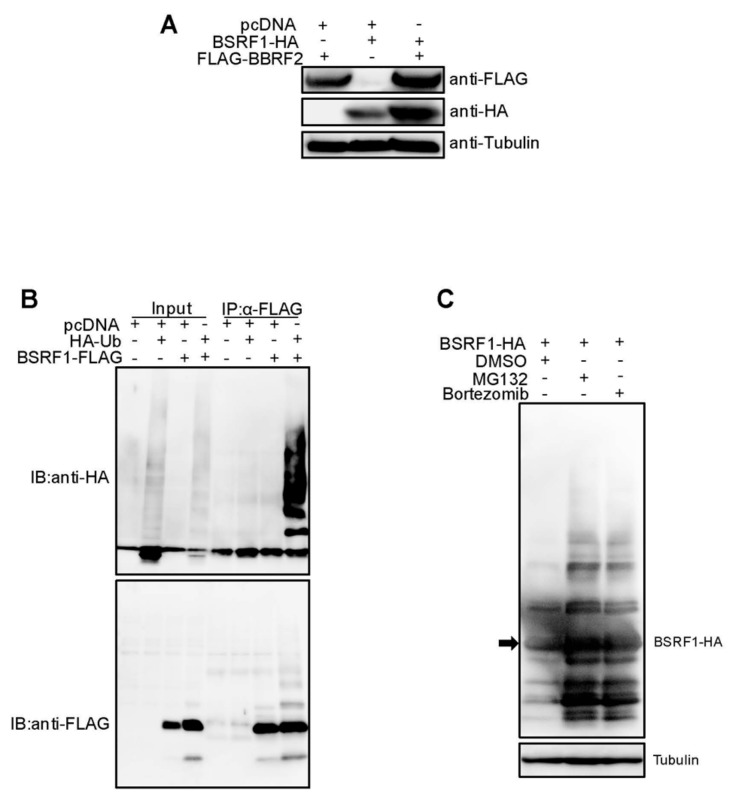
EBV BSRF1, an interaction partner of BBRF2, was protected from ubiquitin/proteasome-dependent degradation by BBRF2. (**A**) Overexpression of the EBV BBRF2 in HEK293T increased the BSRF1 protein level. Cells were transfected with the indicated plasmids and subjected to immunoblot analysis on the following day. (**B**) Ubiquitination of BSRF1. HEK293T cells were transfected with the plasmids for HA-tagged ubiquitin (HA-Ub) and/or FLAG-tagged BSRF1 (BSRF1-FLAG). After one day, a portion of the cell lysate was directly subjected to immunoblotting (input), while the remainder was subjected to immunoprecipitation using an anti-FLAG antibody followed by immunoblotting (IP, α-FLAG). (**C**) Effect of MG132 and Bortezomib on BSRF1 protein. HEK293T cells were transfected with BSRF1-HA expression plasmids and incubated for 24 h in the presence of MG132, bortezomib, or vehicle (dimethyl sulfoxide). Cellular proteins were harvested and subjected to immunoblotting using anti-HA and anti-tubulin antibodies.

The BSRF1 protein level in *BBRF2*-knockout virus-infected Akata cells was lower than that in wild-type-infected cells (Figure 3B). Moreover, the exogenous expression of BBRF2 in knockout virus-infected cells restored the expression of BSRF1 (Figure 4A). Because BSRF1 is an interacting partner of BBRF2 [16], it is possible that the association with BBRF2 stabilizes BSRF1. Also, the HSV UL51 protein level is reportedly dependent on UL7 expression [7]. Therefore, we examined whether BBRF2 overexpression increased the exogenous expression of BSRF1 (Figure 7A). The HA-tagged BSRF1 protein level was low after single transfection and was increased by coexpression of BBRF2 (Figure 7A). Furthermore, BSRF1 protein was polyubiquitinated (Figure 7B), and the BSRF1 level was restored by the proteasome inhibitors, MG132 and bortezomib (Figure 7C). These results suggest that BBRF2 stabilizes BSRF1 by protecting it from ubiquitin/proteasome-dependent degradation.

## 4. Discussion

To our knowledge, the function of the *BBRF2* gene of EBV has not been reported to date. In this study, we analyzed the role of the *BBRF2* gene in the lifecycle of EBV by preparing *BBRF2*-knockout virus using the EBV-BAC (Figure 1 and Figure 2) and CRISPR/Cas9 (Figure 3) systems. The EBV-BAC system is an established recombination technology in bacteria. The major limitation of this system is that, for unknown reasons, the lytic cycle of recombinant virus can be analyzed only in HEK293 cells. As an alternative, we recently established a means of editing the EBV genome using the CRISPR/Cas9 system [23,24]. The Akata-strain EBV edited by our system efficiently infected B cells (Akata cells), and its lytic replication could be induced by the application of an anti-IgG antibody.

The two *BBRF2*-knockout viruses showed the same phenotype, viral protein levels, viral DNA synthesis, and viral progeny production were not affected, but the infectivity of progeny virus was significantly lower (Figure 2 and Figure 3). The reason why the decreased infectious progeny titer was not efficiently restored by exogenous BBRF2 (Figure 4) is unclear, but we speculate that it might be a requirement that the *BBRF2* gene be expressed in a particular stage of the lytic cycle. For example, it may be that BBRF2 must be expressed in the late phase of the lytic cycle, and may inhibit lytic replication if expressed in the immediate-early or early phase. Our results using two knockout strains (B95-8 and Akata), and previous reports on homologous herpesvirus genes, together indicated that BBRF2 augments progeny virus infectivity. Although it is not essential for virus replication, homologs of the EBV *BBRF2* gene are conserved in all herpesviruses (e.g., *UL7* of *HSV*, *UL103* of *CMV*, and *ORF42* of *KSHV*), indicating their functional importance. Despite some sequence variations among virus species, the homologs play a role in efficient production of infectious progeny [7,8,9,10,11,12,13,14].

BBRF2 homolog proteins interact with BSRF1 homologs (UL51 of HSV, UL71 of CMV, and ORF55 of KSHV). We previously reported the BSRF1-BBRF2 association [16]. The intracellular localization of BBRF2 protein changed to the cytoplasm when coexpressed with BSRF1 (Figure 5), as did that of HSV UL7 when coexpressed with UL51 [8]. Furthermore, BSRF1 protein was stabilized by the presence of BBRF2 (Figure 7), as in HSV [7], although it was not examined whether HSV UL51 protein was degraded by the ubiquitin/proteasome pathway.

We also found complex formation of the BBRF2 protein and BGLF2 gene product (Figure 6). The *BGLF2* gene encodes a tegument protein, and is a homolog of the HSV *UL16* and CMV *UL94* genes [5]. Because of the association of BGLF2 with another myristoylated tegument protein, BBLF1 (a homolog of HSV UL11 and CMV UL99), it is also known as myristoylated protein-binding protein (MyrPBP). BGLF2 and BBLF1, like their homologs, are involved in virion maturation and egress, most likely during secondary envelopment [26,31,32,33]. We reported recently that the disruption of the *BGLF2* gene resulted in loss of infectivity of EBV [26]. Therefore, the BBRF2-BGLF2 association may also account for the phenotype of the *BBRF2*-knockout virus, at least in part.

Other tegument proteins have also been implicated in virion maturation and secondary envelopment. For example, the large tegument protein (LTP), BPLF1, and the LTP-binding protein (LTPBP), BOLF1, form a complex and are both involved in virus infectivity [23,34,35]. Homologs of these genes (*UL36* and *UL37* of HSV) reportedly promote intracellular nucleocapsid transport and secondary envelopment [36,37]. In addition, gammaherpesvirus-specific tegument proteins have been linked to virus infectivity. The BRRF2 gene product is localized in the Golgi apparatus and mediates infectious progeny production [38,39], suggesting its involvement in secondary envelopment. Disruption of the *BKRF4* gene resulted in inefficient production of infectious progeny, likely due at least in part to interaction with the *BGLF2* gene product [24]. Therefore, the tegument proteins of herpesviruses form a complicated and sophisticated interaction meshwork between the nucleocapsid and membranous organelle or envelope. Such complexity hinders functional analyses of EBV tegument proteins. The loss of one protein in the meshwork may, to some extent, be compensated for by other protein–protein networks. In fact, no phenotypic change was caused by knockout of *BGLF3.5* (homolog of HSV *UL14*) [40]. Also, the phenotypes of the knockout EBV can be cell type- or virus strain-dependent. For example, disruption of the *BSRF1* gene in EBV-BAC was associated with no phenotype in HEK293, but ablation of the gene product in B95-8 cells resulted in the loss of progeny infectivity [16]. However, in this report, disruption of the *BBRF2* gene was associated with the same phenotype (loss of infectivity) in both EBV-BAC/HEK293 cells and CRISPR/Cas9 system/Akata cells.

In summary, the EBV *BBRF2* gene is important for maximum infectivity. Because this function, and its association with BSRF1 homologs, are conserved among all herpesviruses, it shows promise as a target for antiviral drug development, although further studies are needed to validate this.

## References

[1] Young L.S., Rickinson A.B. (2004). Epstein-Barr virus: 40 Years on. Nat. Rev. Cancer.

[2] Di Napoli A., Al-Jadiri M.F., Talerico C., Duranti E., Pilozzi E., Trivedi P., Anastasiadou E., Alsaadawi A.R., Al-Darraji A.F., Al-Hadad S.A. (2013). Epstein-Barr virus (EBV) positive classical Hodgkin lymphoma of Iraqi children: An immunophenotypic and molecular characterization of Hodgkin/Reed-Sternberg cells. Pediatr. Blood Cancer.

[3] Lieberman P.M. (2015). Chromatin structure of Epstein-Barr virus latent episomes. Curr. Top Microbiol. Immunol..

[4] Murata T. (2014). Regulation of Epstein-Barr virus reactivation from latency. Microbiol. Immunol..

[5] Murata T. (2018). Encyclopedia of EBV-encoded lytic genes: An update. Adv. Exp Med. Biol..

[6] Johannsen E., Luftig M., Chase M.R., Weicksel S., Cahir-McFarland E., Illanes D., Sarracino D., Kieff E. (2004). Proteins of purified Epstein-Barr virus. Proc. Natl. Acad. Sci. USA.

[7] Albecka A., Owen D.J., Ivanova L., Brun J., Liman R., Davies L., Ahmed M.F., Colaco S., Hollinshead M., Graham S.C. (2017). Dual function of the pUL7-pUL51 tegument protein complex in herpes simplex virus 1 infection. J. Virol..

[8] Roller R.J., Fetters R. (2015). The herpes simplex virus 1 UL51 protein interacts with the UL7 protein and plays a role in its recruitment into the virion. J. Virol..

[9] Fuchs W., Granzow H., Klopfleisch R., Klupp B.G., Rosenkranz D., Mettenleiter T.C. (2005). The UL7 gene of pseudorabies virus encodes a nonessential structural protein which is involved in virion formation and egress. J. Virol..

[10] Roller R.J., Haugo A.C., Yang K., Baines J.D. (2014). The herpes simplex virus 1 UL51 gene product has cell type-specific functions in cell-to-cell spread. J. Virol..

[11] Schmitt J., Keil G.M. (1996). Identification and characterization of the bovine herpesvirus 1 UL7 gene and gene product which are not essential for virus replication in cell culture. J. Virol..

[12] Ahlqvist J., Mocarski E. (2011). Cytomegalovirus UL103 controls virion and dense body egress. J. Virol..

[13] Das S., Ortiz D.A., Gurczynski S.J., Khan F., Pellett P.E. (2014). Identification of human cytomegalovirus genes important for biogenesis of the cytoplasmic virion assembly complex. J. Virol..

[14] Butnaru M., Gaglia M.M. (2019). The Kaposi’s sarcoma-associated herpesvirus protein ORF42 is required for efficient virion production and expression of viral proteins. Viruses.

[15] Song M.J., Hwang S., Wong W.H., Wu T.T., Lee S., Liao H.I., Sun R. (2005). Identification of viral genes essential for replication of murine gamma-herpesvirus 68 using signature-tagged mutagenesis. Proc. Natl. Acad. Sci. USA.

[16] Yanagi Y., Masud H., Watanabe T., Sato Y., Goshima F., Kimura H., Murata T. (2019). Initial characterization of the Epstein(-)Barr virus BSRF1 gene product. Viruses.

[17] Nozawa N., Daikoku T., Koshizuka T., Yamauchi Y., Yoshikawa T., Nishiyama Y. (2003). Subcellular localization of herpes simplex virus type 1 UL51 protein and role of palmitoylation in Golgi apparatus targeting. J. Virol..

[18] Kato A., Oda S., Watanabe M., Oyama M., Kozuka-Hata H., Koyanagi N., Maruzuru Y., Arii J., Kawaguchi Y. (2018). Roles of the phosphorylation of herpes simplex virus 1 UL51 at a specific site in viral replication and pathogenicity. J. Virol..

[19] Klupp B.G., Granzow H., Klopfleisch R., Fuchs W., Kopp M., Lenk M., Mettenleiter T.C. (2005). Functional analysis of the pseudorabies virus UL51 protein. J. Virol..

[20] Katsumura K.R., Maruo S., Wu Y., Kanda T., Takada K. (2009). Quantitative evaluation of the role of Epstein-Barr virus immediate-early protein BZLF1 in B-cell transformation. J. Gen. Virol..

[21] Asai R., Kato A., Kato K., Kanamori-Koyama M., Sugimoto K., Sairenji T., Nishiyama Y., Kawaguchi Y. (2006). Epstein-Barr virus protein kinase BGLF4 is a virion tegument protein that dissociates from virions in a phosphorylation-dependent process and phosphorylates the viral immediate-early protein BZLF1. J. Virol..

[22] Daikoku T., Kudoh A., Fujita M., Sugaya Y., Isomura H., Shirata N., Tsurumi T. (2005). Architecture of replication compartments formed during Epstein-Barr virus lytic replication. J. Virol..

[23] Masud H., Watanabe T., Sato Y., Goshima F., Kimura H., Murata T. (2019). The BOLF1 gene is necessary for effective Epstein-Barr viral infectivity. Virology.

[24] Masud H., Watanabe T., Yoshida M., Sato Y., Goshima F., Kimura H., Murata T. (2017). Epstein-Barr virus BKRF4 gene product is required for efficient progeny production. J. Virol..

[25] Murata T., Isomura H., Yamashita Y., Toyama S., Sato Y., Nakayama S., Kudoh A., Iwahori S., Kanda T., Tsurumi T. (2009). Efficient production of infectious viruses requires enzymatic activity of Epstein-Barr virus protein kinase. Virology.

[26] Konishi N., Narita Y., Hijioka F., Masud H.M.A.A., Sato Y., Kimura H., Murata T. (2018). BGLF2 increases infectivity of Epstein-Barr virus by activating AP-1 upon de novo infection. mSphere.

[27] Delecluse H.J., Hilsendegen T., Pich D., Zeidler R., Hammerschmidt W. (1998). Propagation and recovery of intact, infectious Epstein-Barr virus from prokaryotic to human cells. Proc. Natl. Acad. Sci. USA.

[28] Narita Y., Murata T., Ryo A., Kawashima D., Sugimoto A., Kanda T., Kimura H., Tsurumi T. (2013). Pin1 interacts with the Epstein-Barr virus DNA polymerase catalytic subunit and regulates viral DNA replication. J. Virol..

[29] Murata T., Shimotohno K. (2006). Ubiquitination and proteasome-dependent degradation of human eukaryotic translation initiation factor 4E. J. Biol. Chem..

[30] Fossum E., Friedel C.C., Rajagopala S.V., Titz B., Baiker A., Schmidt T., Kraus T., Stellberger T., Rutenberg C., Suthram S. (2009). Evolutionarily conserved herpesviral protein interaction networks. PLoS Pathog..

[31] Starkey J.L., Han J., Chadha P., Marsh J.A., Wills J.W. (2014). Elucidation of the block to herpes simplex virus egress in the absence of tegument protein UL16 reveals a novel interaction with VP22. J. Virol..

[32] Chiu Y.F., Sugden B., Chang P.J., Chen L.W., Lin Y.J., Lan Y.C., Lai C.H., Liou J.Y., Liu S.T., Hung C.H. (2012). Characterization and intracellular trafficking of Epstein-Barr virus BBLF1, a protein involved in virion maturation. J. Virol..

[33] Kopp M., Granzow H., Fuchs W., Klupp B.G., Mundt E., Karger A., Mettenleiter T.C. (2003). The pseudorabies virus UL11 protein is a virion component involved in secondary envelopment in the cytoplasm. J. Virol..

[34] Kumar R., Whitehurst C.B., Pagano J.S. (2014). The Rad6/18 ubiquitin complex interacts with the Epstein-Barr virus deubiquitinating enzyme, BPLF1, and contributes to virus infectivity. J. Virol..

[35] Saito S., Murata T., Kanda T., Isomura H., Narita Y., Sugimoto A., Kawashima D., Tsurumi T. (2013). Epstein-Barr virus deubiquitinase downregulates TRAF6-mediated NF-kappaB signaling during productive replication. J. Virol..

[36] Kelly B.J., Bauerfeind R., Binz A., Sodeik B., Laimbacher A.S., Fraefel C., Diefenbach R.J. (2014). The interaction of the HSV-1 tegument proteins pUL36 and pUL37 is essential for secondary envelopment during viral egress. Virology.

[37] Sandbaumhuter M., Dohner K., Schipke J., Binz A., Pohlmann A., Sodeik B., Bauerfeind R. (2013). Cytosolic herpes simplex virus capsids not only require binding inner tegument protein pUL36 but also pUL37 for active transport prior to secondary envelopment. Cell Microbiol..

[38] Watanabe T., Sakaida K., Yoshida M., Masud H., Sato Y., Goshima F., Kimura H., Murata T. (2017). The C-terminus of Epstein-Barr virus BRRF2 is required for its proper localization and efficient virus production. Front. Microbiol..

[39] Watanabe T., Tsuruoka M., Narita Y., Katsuya R., Goshima F., Kimura H., Murata T. (2015). The Epstein-Barr virus BRRF2 gene product is involved in viral progeny production. Virology.

[40] Watanabe T., Fuse K., Takano T., Narita Y., Goshima F., Kimura H., Murata T. (2015). Roles of Epstein-Barr virus BGLF3.5 gene and two upstream open reading frames in lytic viral replication in HEK293 cells. Virology.

